# Stigmatizing attitudes toward people living with HIV among general adult Thai population: Results from the 5^th^ Thai National Health Examination Survey (NHES)

**DOI:** 10.1371/journal.pone.0187231

**Published:** 2017-11-16

**Authors:** Kriengkrai Srithanaviboonchai, Suwat Chariyalertsak, Jiraluck Nontarak, Sawitri Assanangkornchai, Pattapong Kessomboon, Panwadee Putwatana, Surasak Taneepanichskul, Wichai Aekplakorn

**Affiliations:** 1 Faculty of Medicine, Chiang Mai University, Chiang Mai, Thailand; 2 Research Institute for Health Sciences, Chiang Mai University, Chiang Mai, Thailand; 3 National Health Examination Survey Office, Bangkok, Thailand; 4 Faculty of Medicine, Prince of Songkla University, Songkla, Thailand; 5 Faculty of Medicine, Khon Kaen University, Khon Kaen, Thailand; 6 Faculty of Medicine, Ramathibodi Hospital, Mahidol University, Bangkok, Thailand; 7 College of Public Health Sciences, Chulalongkorn University, Bangkok, Thailand; University of Toronto, CANADA

## Abstract

**Background:**

HIV-related stigma and discrimination is a significant driver of the HIV and AIDS epidemic. UNAIDS encourages all nations to monitor progress toward elimination of this problem. This study measured the level of stigmatizing attitudes toward people living with HIV (PLHIV) among Thai adults in the general population using recommended global tools.

**Methods:**

Data from the 5th National Health Examination Survey, conducted in 2014 were used. The survey utilized six questions recommended by the Global Stigma and Discrimination Indicator Working Group and was administered to participants aged 20–59 years old. All analyses were weighted to take into account of the probability of sampling the same-age Thai population. Factors related to a discriminatory attitude according to UNAIDS, defined as agreed to at least one of the two discriminatory issues, were evaluated using Chi square tested and multivariable logistic regression.

**Results:**

Of the 10,522 respondents, the most prevalent stigmatizing attitude was anticipated stigma (76.9%), followed by perceived stigma (69.2%), fear of HIV infection (57.0%), and social judgment (38.2%). Fifty-eight point six percent had discriminatory attitudes according to the UNAIDS global indicator. Independent predictors were being female (AOR = 1.21: 95% CI 1.14–1.29), aged 20–39 (AOR = 1.19: 95% CI 1.09–1.30) or 50–59 (AOR = 1.18: 95%CI 1.12–1.26), being Muslim (AOR = 2.03: 95%CI 1.55–2.66), earning < 10,000 Baht/month (AOR = 0.93: 95%CI 0.88–0.99), and living in the Northeast (AOR = 1.67: 95%CI 1.39–2.00) or in Bangkok (AOR = 1.73: 95%CI 1.45–2.07).

**Conclusions:**

More than half of the general adult Thai population had stigmatizing attitudes toward PLHIV. The study provided valuable baseline information which could be used as comparison for follow-up surveys with other countries. Interventions to improve Thai society’s knowledge and attitudes toward HIV/AIDS are urgently needed.

## Introduction

HIV/AIDS-related social stigma and discrimination has many serious consequences, both biomedical as well as probable serious psychosocial consequences [1]. Fear of possible HIV stigma prohibits persons who are at risk or in doubt of HIV infection from undergoing HIV testing [2]. This fear may be well-founded as a large proportion of people living with HIV (PLHIV) have avoided or delayed getting the necessary care and treatment due to HIV-related discriminatory acts [3] and fear of lack of privacy and confidentiality of their HIV status at the health care facilities [4]. This phenomenon could further fuel the HIV epidemic since it reduces HIV testing uptake and keeps undiagnosed HIV positive people from receiving needed care.

A qualitative study conducted in Liuzhou, China among PLHIV and health staff found that PLHIV are stigmatized by their family and also face discrimination at work being fired once their HIV-positive status was known [5]. Family members of PLHIV are also targets of HIV-related stigma. A cross-sectional survey on perceived discrimination conducted among 451 PLHIV and 292 caregivers in Haiti found that 32% of children experienced discrimination [6]. These situations demonstrate how the rights of PLHIV and their families have been violated due to stigma.

Social stigma against key populations at higher risk of HIV infection has been shown to be a barrier to needed HIV related health services. A study among 305 men who have sex with men (MSM) and transgender women (TGW) from New York City found that anticipated HIV stigma negatively impacted the likelihood of HIV testing [7]. Seventy-four percent of drug users who participated in methadone maintenance treatment (MMT) in Vietnam reported experiencing HIV-related stigma and discrimination from others [8]. There is also evidence that HIV stigma is prevalent among female sex workers [9, 10] as well as migrant workers [10]. In recognition of this serious problem, UNAIDS encouraged countries to reduce HIV stigma and discrimination as a critical part of national AIDS programs [11] and has defined HIV stigma as a key issue for ending the AIDS epidemic by 2030 [12].

The HIV epidemic in Thailand has been more severe than in its neighboring countries and reached its peak in early 1990s [13]. Thailand’s decisive response to the epidemic has prevented massive needless morbidity and mortality [14]. Recently, Thailand became the first Asian country to achieve the World Health Organization's targets for the elimination of mother to child HIV transmission [15]. However, the latest statistics have affirmed that the country’s progress in addressing the HIV problem has hit a bottleneck as the HIV incidence among men who have sex with men, an important key population at higher risk of HIV infection, is not declining significantly [16]. Evidence has also shown that HIV-related stigma and discrimination is an important barrier to HIV testing among MSM [17, 18] and TGW [18]. Most PLHIV are still entering care late. According to the National AIDS Program, approximately 80% of new HIV cases have initial CD4 cell counts less than 350 cells/l and more than half have CD4 counts less than 100 cells/l by the time they initiate antiretroviral treatment [19].

The current HIV situation in Thailand might be explained in part by the existence of HIV-related stigma and discrimination. The stigma index survey conducted by the Thai Network of People Living with HIV/AIDS (TNP+) in 2009 found that 34% of PLHIV respondents said they had been stigmatized against or refused to join social activities and 20% reported that in the last 12 months they were refused health care services due to their HIV-positive status [20]. Previous studies conducted in the country also revealed that HIV stigma could have a variety of negative impacts on PLHIV including delayed access to care [21], social isolation [22] and depression [23]. A qualitative study found that anticipated HIV stigma in the form of fear of disclosure of HIV-positive status led to poor antiretroviral treatment adherence due to missed medication doses [24]. Evidence also supported that key populations in Thailand were discriminated against. Forty-three percent of MSMs and TGs recruited from gay entertainment venues and community-based organizations in Bangkok and Chiang Mai reported that they experienced discrimination from health staff [25]. For people who inject drugs, it was found that persons who were refused medical care were more likely to avoid health care services [26].

One way to categorize HIV stigma and discrimination is to classify it according to the population of interest. This comprises HIV stigma and discrimination in PLHIV, key populations at higher risk of HIV infection, healthcare workers, policy and law makers, and the general population. HIV stigma and discrimination in each population has its own meaning, function, and significance in the broader paradigm of the HIV stigma dynamic.

The level of HIV stigma and discrimination in the general population of a particular society is significant and has two implications. First, understanding HIV stigma and discrimination in the general population allows health professionals to ‘know your epidemic’ as it reflects the collective thoughts of the public. Earnshaw and Chaudoir [27] elaborated that stigma mechanisms manifested in HIV uninfected individuals (in this case the general population) can impact societal outcomes. Negative attitudes and discriminatory acts against PLHIV in the community would be driven further if the level of HIV stigma and discrimination in the general population is high since people’s thoughts and behaviors tend to be shaped by the actions of the majority [28]. Second, it allows health professionals to ‘know your response’. Countries need valid data on societal level HIV stigma from population surveys to inform programmatic efforts and evaluate intervention outcomes. Repeated measures of this indicator would also reveal trends.

A critical review [29] concluded that there was no systematic approach to measuring HIV stigma and discrimination in the general population. Recognizing the need for consistent measurements of HIV stigma, the Global Stigma and Discrimination Indicator Working Group (GSDIWG) was assembled to develop a set of standardized tools to measure this issue. To assess the level of HIV stigma in the general population in particular, the GSDIWG recommended in 2012 a set of questions to evaluate 6 relevant HIV stigma domains which included fear of HIV infection, social judgment, anticipated stigma, perceived stigma, discrimination (outside legal purview), and discrimination (within legal purview) [30]. Among these, the last 2 questions focusing on experienced discrimination were later selected by UNAIDS and adopted as the global indicators. In 2014, countries were asked to incorporate these questions into their population-based surveys such as the Demographic and Health Survey (DHS) and to include the results in the national Global AIDS Response Progress (GARP) report [31].

Thailand is addressing this issue by adopting zero HIV stigma and discrimination as one of the major goals of the National Strategy on AIDS 2015–2019 [32]. The action plan included an intervention to reduce HIV-related stigma and discrimination in healthcare facilities and a campaign to educate and raise public awareness on the issue. Standardized tools and methods to monitor HIV stigma in health care facilities were also developed [33]. The focus of this study is the national survey on HIV stigma in the general population which was conducted within the national framework for measuring HIV stigma and discrimination. This article describes and discusses the process as well as the relevant results of the 5^th^ National Health Examination Survey (NHES) which included HIV stigma and discrimination questions.

## Materials and methods

### Design and setting

The NHES is a Thai national demographic and health survey that is conducted roughly every 4–5 years. The 5^th^ NHES, which this study is attached to, was carried out during 2013–2014. The survey was conducted under the leadership of the National Health Examination Survey Office with the collaboration of academics from central and regional government universities. The objectives of the survey were to determine the prevalence and risk factors of significant health conditions at the country level as well as the regional level. A four-staged probability sampling technique was used to identify potential participants. These included a sampling of 20 provinces (5 provinces for four regions; north, central, northeast and south) and Bangkok (mandatory site), a sampling of districts within those provinces, a sampling of 540 electoral areas within those districts, and a sampling of 31,700 participants within those electoral areas. The participants were categorized by age into 5 groups; 1–5 years, 6–9 years, 10–19 years, 20–59 years, and 60 years and over. The detailed sampling methodology of the present survey was similar to the previous round which was conducted in 2009 as described elsewhere [34].

### Measurements of HIV stigma

Six questions as suggested in the GSDIWG draft report were used to measure the level of HIV stigma in the general population across 6 domains. These questions were asked to 20–59 years old respondents. These included questions on: 1) anticipated stigma, 2) perceived stigma, 3) fear of HIV infection, 4) social judgment, 5) experienced stigma, and 6) discrimination. All of these questions invited only yes/no responses. The composite Cronbach's alpha of all stigma questions was 0.72. Table 1 details the actual questions asked, the stigma domains measured, and their corresponding scientific meanings. To be sure that all respondents understood the questions correctly, “or AIDS” was added following “HIV” on all questions.

**Table 1:** Questions asked and their corresponded HIV stigma domain and meaning.

Question	HIV stigma domain [30]	Meaning
1. Most people hesitate to take an HIV or AIDS test due to fear of people’s reaction if the test result is positive for HIV.	Anticipated stigma (the fear of negative ramifications should one’s HIV status become known, should one associate with a person living with HIV or should one test positive for HIV)	Agree that people are hesitant to take an HIV test due to anticipation of HIV stigma if the result turns out to be positive
2. People living with or thought to be living with HIV or AIDS lose respect or standing.	Perceived stigma (community members’ perception of stigma that is directed toward people living with HIV by community members)	Agree that people living with HIV are stigmatized or discriminated against if people know or suspect they are HIV positive
3. Do you fear that you could contract HIV or AIDS if you come into contact with the saliva of a person living with HIV?	Fear of HIV infection (fear of infection through casual contact with people living with HIV)	Fear of HIV infection through casual contact with a person living with HIV
4. Do you agree with this sentence?: “I would be ashamed if someone in my family had HIV or AIDS”	Social judgment (shame, blame, prejudice and stereotypes)	Agree with social judgment toward PLHIV
5. You feel too disgusted to buy fresh food or ready-to-eat food from a shopkeeper or vendor whom you know has HIV or AIDS.	Experienced stigma (the experience of discrimination, based on HIV status or association with a person living with HIV or other stigmatized group, that falls outside the purview of the law)	Discriminatory attitude against PLHIV (outside legal purview)
6. You think that children living with HIV or AIDS should not attend the same classroom with other children.	Discrimination (the experience of discrimination that falls within the purview of the law)	Discriminatory attitude against PLHIV (within legal purview)

The last two questions were modified based on recommendations, with ‘yes’ answers representing negative attitudes and ‘no’ answers representing positive attitudes. The outside of legal purview question, ‘Do you feel too disgusted to buy fresh food or ready-to-eat food from a shopkeeper or vendor whom you know has HIV or AIDS?’ was replaced with ‘Would you buy fresh vegetables from a shopkeeper or vendor if you knew that this person had HIV?, and the inside of legal purview question ‘You think that children living with HIV or AIDS should not attend the same classroom with other children’ was replaced with ‘Do you think that children living with HIV should be able to attend school with children who are HIV negative?’

The dichotomous choices of ‘yes’ or ‘no’ were offered to the respondents. Demographics including gender, age, religion, marital status, education, monthly income, urban/rural location (where rural was defined as those living outside of the municipality), and geographical region of the country that they lived in were also asked. Face-to-face interviews by trained interviewers were used to collect the information.

### Statistical methods

All analyses were weighted to take into account the probability of sampling of respondents aged 20–59 years old. Proportions of stigmatizing attitudes were estimated to represent the whole population as well as subgroups according to sex, age-group, religion, marital status, education level, monthly income, urban/rural location, and region. Demographic characteristics of the participants were shown as frequencies and percentages. The proportion of people who answered “yes” which was recognized as stigmatizing attitude of each question was calculated. The respondents who did not answer the questions were excluded from the denominators.

A composite indicator which combined the number of respondents who either answered “yes” to question 5 and/or question 6 (Table 1) as the numerator was computed and used as the main outcome of the study. This composite indicator was recommended by UNAIDS as the global indicator for discriminatory attitude toward PLHIV in the general population. Associations between the demographic characteristics and the main outcome were examined using Chi square tests at the α of 0.05. Bivariate and multiple logistic regression analyses were also performed to identify predictors of discriminatory attitude toward PLHIV according to the global indicator.

### Ethical considerations

All participants provided written informed consent prior to participating in the surveys. The Committee on Human Rights Related to Research Involving Human Subjects, Faculty of Medicine Ramathibodi Hospital, Mahidol University approved the study.

## Results

Of the 10,522 adults aged 20–59 years old who participated in the survey, 59.7% were female, the average age was 44 years old, 93.5% were Buddhists, 73.6% were married and lived with their spouse, 52.0% completed at least primary school, and 49.5% earned less than 10,000 baht (∼300 USD) per month. With regard to the living area, 53.2% lived in urban areas (Table 2).

**Table 2:** Characteristics of the respondents.

Characteristics	n	unweight %	weight %
**Sex**			
- Male	4,239	40.3	48.5
- Female	6,283	59.7	51.5
**Age (years old)** **mean = 44.0**			
- 20–39	3,280	31.2	42.3
- 40–49	3,450	32.8	28.4
- 50–59	3,792	36.0	29.4
**Religion**			
- Buddhist	9,821	93.5	93.7
- Islam	617	5.9	5.8
- Christian	65	0.6	0.5
*- Other*	*3*	*-*	*-*
*- Not answered*	*16*	*-*	*-*
**Marital status**			
- Single	1,674	16.1	19.4
- Couple	7,764	73.6	72.8
- Widow / divorce / separated	995	9.5	7.9
*- Others*	*89*	*-*	*-*
**Education**			
- Not study/primary school	5,422	52.0	49.3
- Secondary / vocational	3,113	29.9	32.6
- Certificate or higher	1,889	18.1	18.1
*- Others + not answered*	*98*	*-*	*-*
**Monthly income (baht)**			
- < 10,000	4,471	49.5	50.4
- ≥ 10,000	4,564	50.5	49.7
*- Others + not answered*	*1*,*487*	*-*	*-*
**Living area**			
- Urban	5,602	53.2	45.7
- Rural	4,920	46.8	54.3
**Region**			
- North	2,323	22.1	16.9
- Central	2,603	24.7	29.4
- Northeast	2,203	20.9	26.2
- South	1,848	17.6	13.5
- Bangkok	1,545	14.7	14.0
**Total**	**10,522**	**100.0**	**100.0**

When ranking the proportion of negative attitude responses to all 6 questions from the highest to the lowest; 76.9% had anticipated stigma, 69.2% had perceived stigma, 57% had fear of HIV infection, 52.1% would practice discrimination, 37.6% expressed social judgment and 24% want to keep HIV-positive children separate from other children. With regard to the UNAIDS global indicator for discriminatory attitude, 58.6% gave negative attitude answers to either question 5 or question 6 (Table 3).

**Table 3:** Percentage and number of the respondents who had negative attitudes according to particular HIV stigma domains.

Domain	The question	Answered ‘Yes’		Did not answer n (%)
		Weight %	n/N	
Anticipated stigma	1. Most people hesitate to take an HIV or AIDS test due to fear of people’s reaction if the test result is positive for HIV.	76.9	8,006/10,464	58 (0.6)
Perceived stigma	2. People living with or thought to be living with HIV or AIDS lose respect or standing.	69.2	7,211/10,461	61 (0.6)
Fear of HIV infection	3. Do you fear that you could contract HIV if you come into contact with the saliva of a person living with HIV?	57.0	6,031/10,458	64 (0.6)
Social judgment	4. Do you agree with this sentence?: “I would be ashamed if someone in my family had HIV or AIDS”	38.2	3,931/10,459	63 (0.6)
Experienced stigma	5. You feel too disgusted to buy fresh food or ready-to-eat food from a shopkeeper or vendor whom you know has HIV or AIDS. (D1)	52.1	5,429/10,465	57 (0.5)
Discrimination	6. You think that children living with HIV or AIDS should not attend the same classroom with other children. (D2)	23.7	2,512/10,455	67 (0.6)
UNAIDS global indicator for discriminatory attitudes toward PLHIV (answered “yes” to either question 5 and/or question 6)		58.6	6,108/10,451	71 (0.7)

When looking at the associations between the demographic characteristics and the UNAIDS global indicator for discriminatory attitude, it was found that there were significant differences between genders (p < 0.001), respondents age groups (p < 0.001), monthly income categories (p = 0.010), and region (p < 0.001). However, the level of discriminatory attitudes did not vary by religion, marital status, education level, or residing in an urban or rural area (Table 4).

**Table 4:** Relationship between characteristics of the respondents and negative attitudes according to particular HIV stigma domains.

Characteristics	weight % of negative attitudes													
	Anticipated Stigma		Perceived Stigma		Fear of HIV Infection		Social Judgment		Discrimination					
									D1		D2		Global indicator (D1 and/or D2)	
	%	p	%	p	%	p	%	p	%	p	%	p	%	p
**Gender**														
- Male	77.1	0.518	70.7	<0.001	53.9	<0.001	42.0	<0.001	49.5	<0.001	24.3	0.061	56.7	<0.001
- Female	76.8		67.8		59.9		34.7		54.6		23.2		60.4	
**Age (years old)**														
- 20–39	78.7	<0.001	72.4	<0.001	59.3	<0.001	36.5	<0.001	54.8	<0.001	22.0	<0.001	60.3	<0.001
- 40–49	76.4		67.0		53.5		36.7		48.4		22.0		55.3	
- 50–59	74.9		66.9		57.1		42.3		51.9		27.9		59.2	
**Religion**														
- Buddhist	76.6	0.007	68.7	0.003	56.1	0.005	37.1	0.007	51.6	0.039	22.8	0.010	57.8	0.030
- Muslim	83.4		77.2		71.0		56.9		61.9		38.7		71.2	
- Christian	63.5		80.4		62.3		26.4		52.2		16.5		60.6	
**Marital status**														
- Single	76.9	0.001	71.3	<0.001	57.6	0.675	35.7	<0.001	52.3	0.677	25.1	0.095	60.0	0.213
- Couple	76.5		68.4		56.9		39.6		52.1		23.3		58.3	
- Widow / divorce / separated	80.8		73.2		57.0		33.5		52.8		24.8		58.6	
**Education**														
- Not study / primary school	75.1	<0.001	66.7	<0.001	57.9	0.010	42.0	<0.001	50.9	0.014	27.3	<0.001	58.1	0.365
- Secondary / vocational	79.2		70.6		55.2		34.7		53.8		20.3		59.3	
- Certificate or higher	78.1		74.0		57.3		34.5		52.8		20.1		58.8	
**Monthly income (baht)**														
- < 10,000	76.6	0.226	67.3	0.029	57.7	0.001	40.0	0.003	52.1	0.118	25.1	<0.001	58.8	0.010
- ≥ 10,000	77.4		69.9		54.7		36.7		50.7		20.9		56.4	
**Living area**														
- Urban	77.3	0.278	71.0	0.005	56.2	0.094	35.3	<0.001	52.9	0.274	22.4	0.002	59.1	0.342
- Rural	76.6		67.8		57.7		40.9		51.5		24.8		58.1	
**Region**														
- North	73.7	<0.001	59.1	0.005	51.2	<0.001	33.0	<0.001	46.1	<0.001	23.0	<0.001	52.8	<0.001
- Central	74.7		70.0		53.2		31.7		45.9		20.5		53.0	
- Northeast	78.9		68.3		59.1		44.8		58.9		25.7		64.6	
- South	80.8		73.5		64.2		45.8		50.6		28.8		58.0	
- Bangkok	77.7		77.4		60.9		38.6		61.3		22.6		66.4	

Bivariate and multivariate analysis of factors associated with the UNAIDS’s global indicator on discriminatory attitudes toward PLHIV showed similar results. Independent predictors of discriminatory attitudes included being female (AOR = 1.21: 95% CI 1.14–1.29), being aged 20–39 (AOR = 1.19: 95% CI 1.09–1.30) or 50–59 (AOR = 1.18: 95%CI 1.12–1.26) as compared to those aged 40–49, being Muslim compared to Buddhist (AOR = 2.03: 95%CI 1.55–2.66), having an income of < 10,000 Baht/month (AOR = 0.93: 95%CI 0.88–0.99), and living in the Northeast (AOR = 1.67: 95%CI 1.39–2.00) and Bangkok (AOR = 1.73: 95%CI 1.45–2.07) compared to living in the North (Table 5).

**Table 5:** Bivariate and multivariate analysis of factors associated with the UNAIDS’s global indicator on discriminatory attitude toward PLHIV.

Independent variables	Crude OR (95% CI)	Adjusted OR (95% CI)
**Gender**		
- Male	1	1
- Female	1.17 (1.10–1.24)*	1.21 (1.14–1.29)*
**Age**		
- 40–49	1	1
- 20–39	1.23 (1.14–1.33)*	1.19 (1.09–1.30)*
- 50–59	1.17 (1.11–1.24)*	1.18 (1.12–1.26)*
**Religion**		
- Buddhist	1	1
- Muslim	1.81 (1.42–2.31)*	2.03 (1.55–2.66)*
- Christian	1.12 (0.79–1.59)	1.26 (0.91–1.75)
**Marital status**		
- Single	1	n/a
- Couple	0.93 (0.87–1.00)	
- Widow / divorce / separated	0.95 (0.86–1.05)	
**Education**		
- Not study / primary school	1	n/a
- Secondary / vocational	1.05 (0.98–1.13)	
- Certificate or higher	1.03 (0.95–1.12)	
**Income (Baht/month)**		
≥ 10,000	1	1
< 10,000	0.91 (0.85–0.97)*	0.93 (0.88–0.99)*
**Living area**		
- Urban	1	n/a
- Rural	0.96 (0.88–1.05)	
**Region**		
- North	1	1
- Central	1.01 (0.85–1.19)	0.99 (0.84–1.17)
- Northeast	1.63 (1.36–1.95)*	1.67 (1.39–2.00)*
- South	1.24 (0.93–1.64)	1.00 (0.81–1.24)
- Bangkok	1.77 (1.48–2.12)*	1.73 (1.45–2.07)*

*statistical significance

## Discussion

To our knowledge, this article is the first to report the results of a national survey on HIV stigma in the general population following the global recommendation. Fifty-nine percent of Thai adults had discriminatory attitudes toward PLHIV based on the UNAIDS definition. The rate of discrimination in Thailand was higher than what was found in China [35], Hong Kong [36], Kenya [37], and Vietnam [38]. However, direct comparisons between these studies and ours is not possible due to discrepancies in the methodologies and measurements used.

This study found that females were more likely than males to have stigmatizing thoughts. One explanation could be that family welfare is primarily considered the woman’s responsibility and this may lead females to be more concerned about food and school safety. Stigmatizing thoughts among females may also be driven by a higher level of fear of HIV infection as evident in this study. People aged 40–49 had lower discriminatory attitudes toward PLHIV as compared to younger (20–39) and older (50–59) age groups. This might be because people in this age group entered adolescence and young adulthood at the peak of Thailand’s HIV epidemic in the early 1990’s [39]. Direct experience of the loss of loved ones to AIDS and exposure to extensive education at that time might have fostered greater sympathy toward PLHIV and explain the lower discriminatory attitude among this group. Our study confirmed that HIV stigma and discrimination is more conspicuous in Muslim cultures. This may be due to religious beliefs regarding sex acts deemed unacceptable and drug-related practices. [40] Further investigation is needed to identify the explanation and the means to mitigate the problem specifically among Thailand’s Islamic communities. Findings that discriminatory attitudes were prevalent in Bangkok and in the Northeast highlight prioritized geographical areas for intervention if resources are limited. Residing in the south, where most Thai Muslims live, was not linked to discriminatory attitudes toward PLHIV in the multivariate model. This result demonstrated the independent effect of these predictor variables toward the outcome and that they should be targeted separately.

When looking into each question that comprised the global composite indicator, 52% of the respondents did not want to buy food from an HIV-positive vendor. This figure was higher than the 35% indicated in the Multiple Indicator Cluster survey among women in Vietnam [38]. Roughly a quarter of general Thai adults thought that children living with HIV or AIDS should not share classrooms with other children. While this represented the smallest proportion of discriminatory attitudes out of all 6 questions, the significance is high since it was the only measurement of discrimination in its strictest meaning (discrimination within legal purview) and affected children which are a vulnerable population.

Anticipated stigma and perceived stigma were the other two manifestations of HIV-related stigma that were measured in this study. The fact that more than three-quarters of Thai adults were hesitant to take an HIV test due to anticipation of stigma is worrisome since HIV testing is the entry point for the needed interventions for both HIV-negative and HIV-positive testers. ‘Treatment as prevention’ [41], the well-recognized strategy to ending AIDS, would not work if most people worry about the stigma they would face if they were HIV-positive. Perceived stigma refers to the degree to which individuals expect PLHIV to experience prejudice and discrimination from others. In other words, it reflects the level of HIV stigma and discrimination in society. This indicator was the second most prevalent item (69.2%) in our survey.

Two drivers of HIV-related stigma and discrimination were measured in this study, fear of HIV infection through casual contact with PLHIV and social judgment toward PLHIV. The findings revealed that 57.0% of Thai adults still held misconceptions about HIV transmission that led to fear of acquiring HIV through casual contact with PLHIV. This issue should be addressed in an education campaign to reduce stigmatizing attitudes among the Thai population. The prevalence of shame in having a PLHIV family member was 38.2%. In all, the presence of these types of stigma highlight the societal barriers to HIV testing for those at risk for HIV and to treatment and disclosure of status for the HIV-positive.

In the past, many countries measured HIV stigma in the general population by either conducting separate surveys [35–37], integrating relevant questions into the DHS, AIDS Indicator Survey (AIS) [42] or Multiple Indicator Cluster surveys [38]. However, the questions and methodologies used varied and a composite indicator to inform the overall situation could not be drawn. The current study demonstrated how Thailand adopted standardized monitoring tools to monitor progress toward reducing discriminatory attitudes as recommended by GSDIWG and UNAIDS. The methodology and findings of this study can serve as the benchmark for future surveys in other countries.

The study provided valuable information highlighting two major ways that future interventions could reduce HIV-related stigma among the general Thai population. First, it helped with targeting who (e.g., females) and where (e.g., Bangkok) the campaign should focus. And second, it helped identify the topics to be included in the campaign. The results of this study confirmed the need to educate the public on basic HIV knowledge (e.g., how HIV is spread) as most people still believe that they could contract HIV through casual contact. Many people still held negative attitudes toward HIV as they felt it was shameful to be infected with HIV.

There are ways to reduce HIV stigma in the general public. A study conducted in Northeastern Thailand revealed that community interventions which empower the community are effective on increasing interaction between PLHIV and other community members [43]. A systemic review confirmed that interactions between PLHIV and general publics were effective in improving people’s attitudes [44]. An education campaign to educate the general population about how HIV virus is transmitted is another approach that might help reduce HIV stigma. Another study conducted in Northern Thailand found that inaccurate beliefs about HIV transmission are related to fear and stigmatizing attitudes toward PLHIV [45]. These strategies might be included in a future intervention. The results of the follow-up surveys will also reveal trends and serve as an outcome evaluation of the intervention.

There are some strengths of this study that should be mentioned. As remarked earlier, this is the first survey report on HIV stigma and discrimination in the general adult population using recommended global tools. The overall validity of the study should be sound as it was a sub-study of the NHES which has rigorous methodology and quality control. Like other population-based probability sampling surveys, the results should well represent the characteristics of the intended population, in this case adults in the general population. All figures were weighted to reflect the actual population instead of reporting crude results.

A major limitation of this study is the limited comparability with other surveys and the global indicator since some questions were adapted and the respondent age range (20–59) did not match the age range for the global recommendations (15–49). The study had to rely on data available from the NHES which intended to capture only a broad picture of the Thai population’s health status. Other significant variables with regard to HIV-related stigma and discrimination such as HIV knowledge, sexual orientation and key population status of participants were not measured because of this constraint. Similar to other surveys focused on sensitive social issues, social desirability bias might be an issue, leading to under-reporting of discriminatory attitudes.

## Conclusions

In conclusion, the study revealed that a large proportion of adult Thais in the general population had stigmatizing attitudes toward PLHIV. It also identified particular groups of the population that should be targeted for future interventions. The study provided valuable baseline information that could be compared with follow-up surveys as well as across countries and underlined the need for programs to improve Thai society’s knowledge and attitudes toward HIV/AIDS.

## Supporting information

S1 Dataset (SAV)
